# Elucidating the Neuroprotective Role of PPARs in Parkinson’s Disease: A Neoteric and Prospective Target

**DOI:** 10.3390/ijms221810161

**Published:** 2021-09-21

**Authors:** Tapan Behl, Piyush Madaan, Aayush Sehgal, Sukhbir Singh, Neelam Sharma, Saurabh Bhatia, Ahmed Al-Harrasi, Sridevi Chigurupati, Ibrahim Alrashdi, Simona Gabriela Bungau

**Affiliations:** 1 Chitkara College of Pharmacy, Chitkara University, Punjab 140401, India; piyushmadaan4811@gmail.com (P.M.); aayushshehgal00@gmail.com (A.S.); sukhbir.singh@chitkara.edu.in (S.S.); neelam.mdu@gmail.com (N.S.); 2 Natural & Medical Sciences Research Centre, University of Nizwa, Birkat Al Mauz 616, Nizwa P.O. Box 33, Oman; sbsaurabhbhatia@gmail.com (S.B.); aharrasi@unizwa.edu.om (A.A.-H.); 3 School of Health Science, University of Petroleum and Energy Studies, Dehradun 248007, India; 4 Department of Medicinal Chemistry and Pharmacognosy, College of Pharmacy, Qassim University, Buraydah 52571, Saudi Arabia; sridevi.phd@gmail.com; 5 Translational and Clinical Research Institute, Newcastle University, Newcastle-upon-Tyne NE1 7RU, UK; i.alrashdi2@newcastle.ac.uk; 6 Department of Pharmacy, Faculty of Medicine and Pharmacy, University of Oradea, 410028 Oradea, Romania; 7 Doctoral School of Biological and Biomedical Sciences, University of Oradea, 410073 Oradea, Romania

**Keywords:** neurodegenerative diseases, peroxisome proliferator-activated receptors, oxidative stress, mitochondrial dysfunction, Parkinson’s disease, neuroprotection

## Abstract

One of the utmost frequently emerging neurodegenerative diseases, Parkinson’s disease (PD) must be comprehended through the forfeit of dopamine (DA)-generating nerve cells in the substantia nigra pars compacta (SN-PC). The etiology and pathogenesis underlying the emergence of PD is still obscure. However, expanding corroboration encourages the involvement of genetic and environmental factors in the etiology of PD. The destruction of numerous cellular components, namely oxidative stress, ubiquitin-proteasome system (UPS) dysfunction, autophagy-lysosome system dysfunction, neuroinflammation and programmed cell death, and mitochondrial dysfunction partake in the pathogenesis of PD. Present-day pharmacotherapy can alleviate the manifestations, but no therapy has been demonstrated to cease disease progression. Peroxisome proliferator-activated receptors (PPARs) are ligand-directed transcription factors pertaining to the class of nuclear hormone receptors (NHR), and are implicated in the modulation of mitochondrial operation, inflammation, wound healing, redox equilibrium, and metabolism of blood sugar and lipids. Numerous PPAR agonists have been recognized to safeguard nerve cells from oxidative destruction, inflammation, and programmed cell death in PD and other neurodegenerative diseases. Additionally, various investigations suggest that regular administration of PPAR-activating non-steroidal anti-inflammatory drugs (NSAIDs) (ibuprofen, indomethacin), and leukotriene receptor antagonists (montelukast) were related to the de-escalated evolution of neurodegenerative diseases. The present review elucidates the emerging evidence enlightening the neuroprotective outcomes of PPAR agonists in in vivo and in vitro models experiencing PD. Existing articles up to the present were procured through PubMed, MEDLINE, etc., utilizing specific keywords spotlighted in this review. Furthermore, the authors aim to provide insight into the neuroprotective actions of PPAR agonists by outlining the pharmacological mechanism. As a conclusion, PPAR agonists exhibit neuroprotection through modulating the expression of a group of genes implicated in cellular survival pathways, and may be a propitious target in the therapy of incapacitating neurodegenerative diseases like PD.

## 1. Introduction

Parkinson’s disease (PD) is a common, intricate, progressive, multifaceted, and debilitating neurodegenerative disease, which is portrayed by the forfeiture of dopamine (DA) generating nerve cells in the substantia nigra pars compacta (SN-PC). Moreover, a pathogenic feature of PD is the accumulation of protein named α-synuclein in Lewy bodies (LBs) and Lewy neurites pinpointed within the nerve cells [1]. Tremor, bradykinesia, rigor, and postural abnormalities emerge as an integral manifestation associated with PD [2]. In those under the age of 40, PD is exceedingly rare, but it affects nearly 1–2% of people over 60–65 years of age and presents a comparative higher risk of developing PD in people beyond 85 years of age worldwide [3]. The incidence of PD differs among genders, with women exhibiting lesser vulnerability to developing PD than men, because of the neuroprotective outcomes rendered by estrogen in the case of women [4]. Although the exact etiology of PD is unclear, various genetic and environmental factors are believed to play a pivotal role in the progression of the disease [5]. Even though the critical pathways involved in the commencement and progression of PD are still unknown, increased oxidative stress, ubiquitin-proteasome system (UPS) dysfunction, autophagy-lysosome system dysfunction, neuroinflammation and programmed cell death, and mitochondrial dysfunction are presumed to be actively engaged in the pathogenesis of PD [5]. Existing pharmacotherapy can only furnish symptomatic relief, and no treatment has been displayed to halt the disease progression [6].

Peroxisome proliferator-activated receptors (PPARs) are ligand-directed transcription factors belonging to the class of nuclear hormone receptors (NHR), and are actively engaged in the regulation of mitochondrial functioning, inflammatory processes, redox balance, wound healing, and metabolism of blood sugar and lipids [7]. three subtypes of PPARs have been promulgated, viz., PPAR-α, PPAR-β/δ, and PPAR-γ, and are pinpointed in various body regions. These subtypes have been reported to partake in the modulation of inflammatory processes, and regulation of numerous incapacitating neurodegenerative conditions [8]. PPARs are activated with the aid of tiny lipophilic molecules, which subsequently form heterodimers with their partner named retinoid X receptors (RXR) in order to carry out comprehensive cytoplasmic stimulation. This heterodimer interacts with DNA sequence elements termed peroxisome proliferator response elements (PPREs) so as to modulate the transcription of genes that are actively engaged in various biological activities, such as inflammatory processes, insulin sensitization, and neuronal protection [9]. Several PPAR agonists, for instance, pioglitazone, rosiglitazone, fenofibrate, benzafibrate, and others have been shown to safeguard nerve cells from oxidative stress, inflammation, and programmed cell death in PD and other incapacitating neurodegenerative diseases and are enumerated in this review [10]. Moreover, several studies have linked the regular use of PPAR-activating non-steroidal anti-inflammatory drugs (NSAIDs) (ibuprofen, indomethacin) [11,12], leukotriene receptor antagonists (montelukast) [13], and physical exercise [14] to the de-escalation of neurodegenerative conditions. Owing to the rising complexity in the treatment of neurodegenerative diseases, PPARs have received a considerable importance of late.

The current review aims to highlight the investigations elucidating the mode of action and neuroprotective outcomes of PPAR agonism in numerous experimental models experiencing PD. The result is an informative work that should be very useful for future publications in the field of PD.

## 2. Cellular Influences of PPARs

The peroxisomes, otherwise known as microbodies, are sub-cellular structures spotted inside the building blocks of nearly all plants and animals that carry out varied biotransformation activities, such as fatty acid (FA) oxidation, hydrogen peroxide (H_2_O_2_)-reliant respiration, and metabolism of lipids [15]. PPARs are ligand-directed transcription factors that pertain to the class of thyroid, steroid, and retinoid receptors and often referred to as NHR [16,17]. These receptors play a pivotal role in the modulation of numerous genes, and biochemical pathways, such as the modulation of mitochondrial operation and redox equilibrium, for instance [18,19]. Up to the present, the trio, particularly PPAR-α (NR1C1), PPAR-β/δ (NR1C2), and PPAR-γ (glitazone receptor/NR1C3) receptor subtypes have been recognized, which are coded by specific genes positioned on the 22, 6, and 3 chromosomes of human beings, consecutively [20]. The three subtypes are effectively modulated via an additional group of genes designated as transcriptional co-activators [21]. Typically, small lipophilic molecules trigger the activation of PPARs, which then form heterodimers with their partner, namely RXR for their exhaustive cytoplasmic stimulation [22]. The amino (N)-terminus ligand self-reliant trans-activation portion, a deoxyribonucleic acid (DNA) interacting portion, and a carboxy (C)-terminus ligand-interacting portion and ligand-based actuation portion are entirely retained within the configuration of these varied subtypes of PPARs [23]. This C-terminus portion of the receptor is presumed to be actively engaged in creating heterodimers with its mate, the RXR. Within the modulatory portion of their target genes the RXR or PPAR heterodimer selectively interacts with DNA sequence elements referred as PPREs in order to control the transcriptional operation. 

The stimulation of the plethora of gene cascades implicated in a wide range of body functions takes place following the selective interaction of PPAR with DNA sequences [24]. The interaction of PPAR as well as RXR heterodimers with the co-repressor complexes and the repression of transcription of genes occur during the non-appearance of ligands [25]. In contrast, the presence as well as interaction of natural (FA, and related compounds), and synthetic ([4-[3-(4-Acetyl-3-hydroxy-2-propylphenoxy)propoxy]phenoxy]acetic acid (L-165041), 2-[2-methyl-4-[[4-methyl-2-[4-(trifluoromethyl)phenyl]-1,3-thiazol-5-yl]methylsulfonyl]phenoxy]acetic acid (GW-501516), glitazones/thiazolidinediones (TZDs), and fibrates) and ligands provokes a configurational transition inside the PPAR, prompting co-repressor amino acid chain to detach and the co-activator amino acid chain to engage in order to elevate target gene transcription [16,23,26]. Several studies have revealed that certain NSAIDs, including indomethacin, ibuprofen, naproxen, and fenoprofen also stimulate PPAR-α, and PPAR-γ, relying upon their affinity to interact with PPAR [27,28]. 

The varied subtypes of PPAR are produced via a single PPAR gene, and their functioning is ascertained by the body tissues within which they remain embodied. These subtypes partake in distinct biological, therapeutic, and molecular processes, such as the modulation of thermogenesis, transcription, the metabolism of lipids, and mitochondrial FA β-oxidation, as well as display discrete particularities for ligands, relying upon their variable positioning and genes of target within the body tissues [15,16]. These subtypes differ in their extent of activity and distribution among various body organs and tissues. PPAR-α is chiefly found in the liver, and inferiorly in the muscle, bone, cardiac and renal region, and participate in facilitating FA usage and catabolism through the overexpression of genes implicated in the conveyance of FA, the metabolism of lipids, and peroxisomal and mitochondrial β-oxidation of FA [15,29,30]. Both natural FA and synthetic ligands (for instance, fibrates-antihyperlipidemic agents) hold the promise to activate PPAR-α [31]. PPAR-β/δ is widely displayed across the entire body, and is actively engaged in controlling the metabolism of blood sugar and lipids [15,31]. PPAR-γ is present in nearly all body tissues, including the large intestine, muscle, spleen, pancreas, the cardiac and renal regions, adipose tissue, macrophages, and endothelial cells, where it actively participates in the metabolism of blood sugar, the regulation of storage of FA, cell enlargement, adipogenesis, and insulin sensitivity [15,32]. Activation of PPAR-γ is triggered by a number of natural ligands and also the synthetic ligands (TZDs) [31]. 

**Figure 1:**
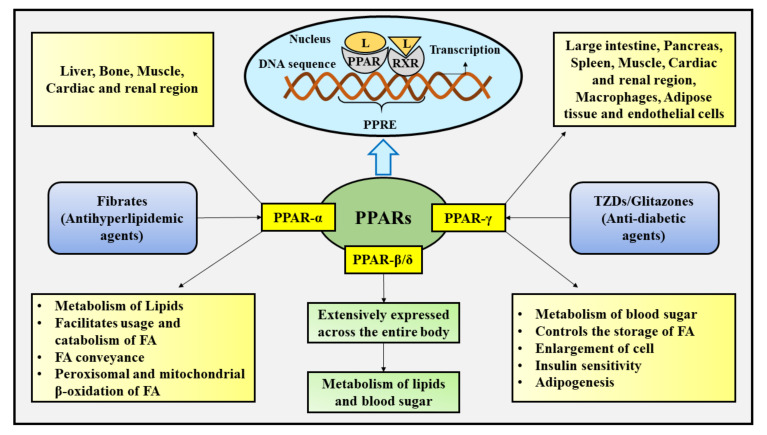
Location, ligand-based activation, functions, and transcriptional activation of PPARs. FA, fatty acids; L, ligand; PPARs, Peroxisome proliferator-activated receptors; RXR, retinoid X receptors; DNA, deoxyribonucleic acid; PPRE, peroxisome proliferator response element; TZDs, thiazolidinediones.

The three subtypes of PPAR are known to exert the modulatory effect on the inflammatory pathways [33]. Besides the modulation of transcriptional action of nuclear factor kappa B (NF-κB), additional transcriptional factors associated with the inflammatory reactions are also controlled by PPARs as well as additional nuclear receptors. It involves the control of numerous transcriptional factors, namely activating transcription factor-1 (ATF-1), activating transcription factor-4 (ATF-4), and signal transducer and activator of transcription (STAT), along with the control of levels of various inflammatory mediators, for instance, cyclooxygenase-2 (COX-2), and nitric oxide (NO) synthase [33]. It has been reported that PPARs modulate the process of inflammation via various and numerous pathways. At the initial stage, PPAR contends with NF-κB to interact with a protruded collection of co-activators, in particular the cyclic AMP-response element binding protein (CREB), consequently suppressing the inflammatory reaction effectuated by NF-κB [33,34]. Further, the PPAR binds straightforward to the p50, REL-associated protein (RELA)/p65, and IkappaB alpha (IκBα), and results in the inhibition of ability of NF-κB to interact with DNA [33]. In addition, the PPAR suppresses NF-κB as well as activator protein-1 (AP-1) signal-reliant transcriptional stimulation of genes associated with inflammation via a well-renowned process termed as trans repression, by straightforward protein-protein interaction with promoter-interacted transcription factors as well as via the prevention of signal-reliant co-repressor complex elimination [26,33,34]. Figure 1 depicts the location, ligand-based activation, functions, and transcriptional activation of PPARs.

Currently there has been explosion in the exploration of effect of PPARs on the functioning of mitochondria. Pioglitazone, a PPAR-γ agonist, belonging to the class of TZDs has been reported to elevate the intake of oxygen (O_2_), mitochondrial DNA (mtDNA) levels, as well as the activity of several factors associated with the expansion and division of already existing mitochondria (mitochondrial biogenesis), namely mitochondrial transcription factor A (Tfam) and PPAR-gamma co-activator-1 alpha (PGC-1α) within the adipose tissue under the skin, and the neuronal NTERA-2 (NT2) cell line of humans [35,36,37]. In addition, pioglitazone also enhances blood sugar metabolism and mitochondrial activity within astroglia [38]. Additionally, MitoNEET, an iron-sulfur (2Fe-2S) comprising protein located externally to the mitochondrial membrane that plays a crucial role in modulating the oxidative capability, is balanced with the aid of pioglitazone [39,40]. Rosiglitazone, an aminopyridine pertaining to the TZDs category of drugs has been reported to induce the consumption of blood sugar and mitochondrial biogenesis within the brain of the experimental mouse model [41]. Moreover, glitazones raise mitochondrial membrane potential, and thereby assist in safeguarding the cells from undergoing programmed cell death subsequent to the elimination of growth factors [42]. The subtypes of PPAR actively participate in modulating evolution, inflammatory processes, healing of wounds, operation of mitochondria, tissue differentiation, and metabolism of blood sugar and lipids [43,44]. Owing to the effective participation of PPARs in various processes (such as mitochondrial biogenesis, inflammatory processes, metabolism of blood sugar and lipids, and cell differentiation) they furnish safeguarding outcomes in fatty liver disease [45], ischemic stroke [46], cancer [47], tumors [47,48], cardiac disorders [49], and type II diabetes [50].

Numerous studies have revealed that PPARs are also expressed in central nervous system (CNS) nerve cells and astroglia, which incentivized the researchers to scrutinize the PPAR agonists for their neuroprotective activity in a variety of neurodegenerative diseases [31,51]. PPAR agonists have been demonstrated to exert a protective action on tissues in the peripheral region, against oxidative injury, inflammatory processes, and programmed cell death. Moreover, these agonists have been revealed to possess a neuroprotective action in various CNS diseases [31,51]. Following short-term localized ischemia in rodents, pioglitazone and rosiglitazone restrain neuronal injury and inflammatory processes, thereby exhibiting neuroprotective action [52,53]. It has been reported that rosiglitazone, by elevating the expression of the B-cell lymphoma 2 (Bcl-2) protein defends the nerve cells of spinal ganglion and hippocampus from amyloid-beta (Aβ)-prompted mitochondrial injury [54]. Pioglitazone and rosiglitazone effectively restrain myelin deprivation, pain associated with neuropathy, neuronal injury, and inflammatory processes, and upgrade locomotor recuperation following damage to the rodent spinal column [55,56]. In cortical nerve cells and mixed glia cultures, agonism of PPAR-γ has been reported to elicit a pathway for the removal of Aβ peptide [57]. The PPAR family emerges to be a neoteric, propitious, and prospective target which holds the aptitude to assist in the therapy of neurodegenerative diseases, namely PD [31,58], Alzheimer’s disease (AD) [31,59], Huntington’s disease (HD) [31,60], Amyotrophic lateral sclerosis (ALS) [31,60], and Multiple sclerosis (MS) [61]. 

## 3. Parkinson’s Disease 

The term PD, otherwise known as paralysis agitans, was coined in 1817 by a British physician named James Parkinson [62]. PD is a common, persistent, intricate, progressive, and generally incapacitating neurodegenerative condition that is concerned with numerous motor and nonmotor brain systems, and predominantly affects elderly people [62,63]. The condition is portrayed by the loss of DA producing nerve cells in the SN-PC, culminating in striatal DA deficiency [58,64]. The build-up of α-synuclein protein in LBs and Lewy neurites located within the nerve cells is also regarded as a pathological feature of PD [65,66]. Following AD, PD is considered as the second most prevalent neurological disease closely related to substantial impairment and decline in well-being [64]. The chief manifestations of PD comprise tremor (shaking), rigor (stiffness), bradykinesia (slowness of movement), and postural abnormality (impairment in body posture and balance) [67]. 

In developed regions, the pervasiveness of PD is higher, and it has been proven to elevate with age [68]. PD is extremely uncommon in individuals below 40 years of age, whereas it significantly affects 1–2% of individuals beyond 65 years of age, and 4-5% of individuals beyond 85 years of age globally [69]. The occurrence of PD varies among genders, with women being less susceptible to developing PD in comparison to men, owing to the neuroprotective action exhibited by estrogen in women [70]. 

The underlying cause of PD is still obscure, however numerous genetic and environmental factors are presumed to be implicated in the evolution of the disease [67]. Mutations in genes, such as *ubiquitin C-terminal hydrolase L1*(*UCHL1*) [71], *α-synuclein*(*SNCA*) [72], *leucine-rich repeat kinase 2*(*LRRK2*) [73], *Parkin RBR E3 ubiquitin-protein ligase*(*Parkin*) [74], *PTEN-induced kinase 1*(*PINK1*) [74], *protein deglycase*(*DJ-1*) [75], and *glucocerebrosidase*(*GBA*) [76] can result in the development of PD. Several environmental factors, like exposure to pesticides (rotenone, and paraquat) [77], methanol (CH_3_OH) [78], injury to the head [79], and poisoning of carbon monoxide (CO) [80] are thought to be associated with the development of PD. 

## 4. Etiology of PD

**Figure 2:**
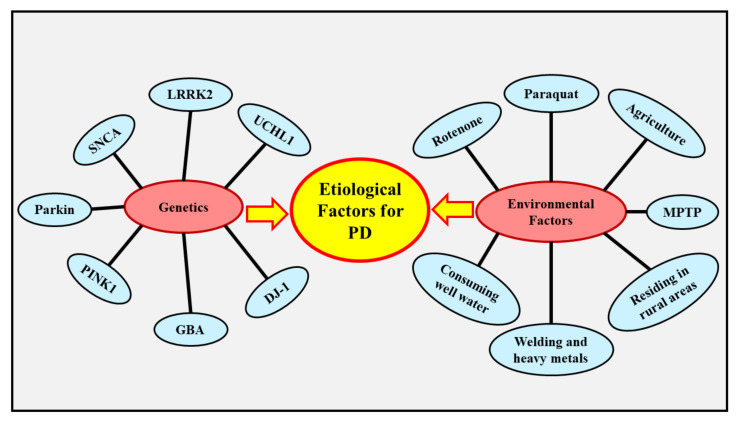
Etiological factors for Parkinson’s disease. UCHL1, ubiquitin C-terminal hydrolase L1; LRRK2, leucine-rich repeat kinase 2; SNCA, α-synuclein; Parkin, Parkin RBR E3 ubiquitin-protein ligase; PINK1, PTEN-induced kinase 1; GBA, glucocerebrosidase; DJ-1, protein deglycase; PD, Parkinson’s disease; MPTP, 1-methyl-4-phenyl-1,2,3,6-tetrahydropyridine.

PD is an intricate and multifaceted condition in which genetic and environmental factors contribute profoundly. The preponderance of patients experience the sporadic (typically delayed commencement) form of PD instead of the familial (typically early commencement) form of PD, and they arise due to genetic, environmental, or both of these factors together. Mutations in genes have been found to be associated with approximately 15% of patients experiencing PD, particularly with the familial form [81]. Age is regarded as a major element of danger for PD, as 60 years of age is the mean age of commencement [82]. The occurrence of the condition escalates with aging, hitting 93.1 (every 100,000 person-years) in those aged 70–79 years [83,84]. There are also cultural diversity differences, with European, South American, and North American countries indicating elevated incidence in comparison to African, Arabic, and Asian countries [85] Figure 2 depicts the factors that contribute to the etiology of PD.

### 4.1. Genetics

Even though PD is predominantly a condition with an unknown cause, around 10–15% of individuals indicate a familial history, and around 5% of cases arise due to genetic inheritance [86]. Mutations in multitudinous genes, such as *UCHL1*, *SNCA*, *LRRK2*, *Parkin*, *PINK1*, *DJ-1*, and *GBA* have been linked to PD [71,72,73,74,75,76].

The *UCHL1* gene, otherwise termed as protein gene product 9.5 (PGP9.5), appears to be a physiologically feasible predisposing gene for PD [71,87]. The articulation of *UCHL1* is nerve cell specific and pervasive all over the brain, displaying notably robust in situ hybridization findings inside the SN-PC [88]. The *UCHL1* enciphers a protein which constitutes nearly 1–2 % of the entire soluble protein present within the brain and is often discovered in LBs [89]. It has been proven that *UCHL1* actively participates in ubiquitin-reliant cleavage of proteins/polypeptides via converting large structural repeating units of ubiquitin to a single unit of ubiquitin. In order to undergo degradation via proteasomes, ubiquitin effectuates activation, coupling, and joining with prejudicious proteins. The interruption of the entire ubiquitination as well as proteasomal degradation system and the consequent accumulation of *SNCA* within the cytoplasm has been postulated as an underlying mechanism for PD [90]. The gracile axonal dystrophy (gad) mouse involves the removal of *UCHL1* within the gene, which in turn contributes to usual manifestations associated with neurodegeneration, for instance, deprivation of voluntary muscles balance, dying back type neuronal degeneration [91], and protein deposition in nerve endings [92]. The accumulation of *UCHL1* and its isotypes related to PD, comprising *UCHL1*^S18Y^, and *UCHL1*^I93M^, is escalated within cultured cells, following the suppression of the UPS, thereby demonstrating a potential correlation between PD and UPS [93]. Moreover, mutations in *UCHL1* isotypes, namely *p.I93M, p.E7A*, and *p.S18Y* are strongly linked with tremendous hazard towards PD [87]. These investigations disclose the substantial contribution of mutations in the *UCHL1* gene and its isotypes to the evolution of PD.

The *SNCA* gene ciphers in order to produce a protein named α-synuclein which exists in nerve cells in the vicinity of presynaptic nerves as well as additional types of cells. This protein shares active involvement in synaptic transmission since it effectively controls the quantity and liberation of DA comprising neurotransmitter vesicles [94]. It has been reported that *SNCA* gene mutations can result in the build-up of this protein, which consecutively contributes to the anomalous amassing of DA. This results in making the body capable of splitting the profuse DA, which results in nerve cell death and the emergence of manifestations associated with PD [94].

The sporadic form of PD, which arises beyond 50 years of age, has been linked to *LRRK2* gene mutations [95]. Dardarin, a protein possessing multiple domains, which is encoded by the *LRRK2* gene, has been found to partake in transmission processes essential for protein-protein signaling and the operation of nerve cells [95]. The conformation and activity of dardarin proteins are greatly influenced by *LRRK2* gene mutations. Several researchers have scrutinized and revealed that the dardarin mutant triggers programmed cell death, and its interaction with a protein termed parkin gives rise to an accumulation of cytoplasmic proteins [96]. Mutations in the *LRRK2*gene prompt breakdown and build-up of protein in an aberrant manner [97]. Elevated build-up of cytoplasmic proteins might promote programmed cell death, which in turn results in abnormalities in mobility and coordination that are often noticeable in patients experiencing PD, but the underlying pathways are still obscure [98]. 

The *Parkin*/*PARK2* gene ciphers parkin (protein) that is speculated to direct proteins so as to effectuate breakdown with the aid of enzymes. *Parkin* has also been associated with the breakdown of impaired cell powerhouses/ energy factories (mitochondria). Autosomal recessive, early commencement forms of PD are found to be associated with *PARK2* gene mutations [95]. As a consequence of *PARK2* gene mutations, the parkin protein starts operating abnormally, and it has been noted that this deprivation of the usual functioning of parkin elicits the build-up of inappropriate proteins, which in turn could disrupt DA release and other usual cellular functions [99]. Owing to the profuse presence of parkin within the CNS, its abnormal functioning could result in the deprivation of DArgic nerve cells, which, as a result, contributes to the emanation of manifestations related to PD [98]. In addition, several investigations have reported that mutations in the *PARK2* gene are also associated with diminished functioning of the powerhouse of the cell and elevated susceptibility towards substances that are harmful to the powerhouse of the cell, and in the case that the cells’ powerhouse in DArgic nerve cells is disrupted, it could impair the conveyance of DA, potentially contributing to the manifestation of PD [95]. 

Apart from this, mutations in the *PINK1* gene are actively engaged in precipitating manifestations of PD. It has been elucidated that those mutations in the *PINK1* gene are explicitly related to autosomal recessive, early commencement forms of PD [100]. PTEN, a protein encoded by the *PINK1* gene, is expressed within the cellular energy factories across the body, and is presumed to exert a safeguarding action against oxidative damage [95]. The typical PTEN protein has been reported to suppress programmed cell death, whereas the mutant form of PTEN protein is powerless to suppress programmed cell death, and thereby might give rise to escalated nerve cell destruction.

The *DJ-1* protein, otherwise termed as *PARK7*, which behaves as an antioxidant and safeguards nerve cells against oxidative damage, and restrains the α-synuclein build-up, is ciphered by the *PARK7* gene. It has been elucidated that *PARK7* gene mutations provoke the abnormal operation of *DJ-1*/*PARK7* protein, eventually resulting in the build-up of α-synuclein as well as the accumulation and breakdown of profuse DA [99]. The abnormal operation of *DJ-1*/*PARK7* induces oxidative damage, which consecutively evokes DArgic nerve cell destruction. In each of the aforementioned scenarios, the deprivation of DA is thought to play an integral role in the emergence of manifestations of PD [95].

It has been elucidated that the *GBA* gene ciphers the lysosomal enzyme named β-*GBA*, which effectuates the breakdown of sphingolipid, namely glucosylceramide (GluCer), as a means of producing a pair of components termed glucose (sugar), and ceramide (lipid molecule) [101]. It has been evaluated that nearly 12% of European patients experiencing PD, and 15 to 20% of Ashkenazi Jewish patients experiencing PD, are robustly linked with mutations and variations in the *GBA* gene, creating *GBA* as a critical genetic hazard for PD [102]. Patients who express mutations in the *GBA* gene are at a risk of developing PD earlier in life, as well as exhibiting cognitive disability [101]. In patients with sporadic forms of PD, the functioning of β-*GBA* is greatly diminished within the anterior cingulate cortex (ACC), and substantia nigra (SN) regions of the brain [103,104]. The disabled autophagy-lysosomal pathway (ALP) is presumed to be actively engaged in the α-synuclein build-up in an aberrant manner [101]. It has been reported that α-synuclein builds up and displays LBs attributes in physiological and experimental models possessing knocking down, knocking out or mutations in the β-*GBA*, and is associated with ALP disability [101]. Even though the precise pathway via which deprivation of β-*GBA* participates in the pathophysiology of PD is still poorly understood, it might comprise α-synuclein build-up, diminished lysosomal operation, and endoplasmic reticulum (ER)-related stress [105]. Considering homozygous mutations in the *GBA* gene, GluCer build-up within the lysosomes might provoke lysosomal abnormalities, whereas no such build-up of GluCer has been found in PD brains possessing heterozygous mutations in the *GBA* gene [105].

### 4.2. Environmental Factors

Besides the involvement of genetic factors in the evolution of PD, there are several environmental factors which significantly contribute to PD. The neurotoxin1-methyl-4-phenyl-1,2,3,6-tetrahydropyridine (MPTP), was initially recognized to be related to nigrostriatal degeneration, following the emergence of characteristic manifestations of PD in several individuals upon self-administration of narcotic substances contaminated with MPTP. MPTP is bio transformed into an active toxic metabolite named 1-methyl-4-phenylpyridinium ion (MPP+), which belongs to the family of mitochondrial complex-I suppressors, and is exclusively involved in devastating DArgic nerve cells within the SN [106,107]. The exploration of MPTP as a triggering factor for degeneration within the SN encouraged the postulation that PD might be precipitated by toxic substances present in the environment [108]. 

Thereafter, numerous investigations have revealed the significant relationship between exposure to pesticides and PD, particularly a single case-referent study demonstrating a strong correlation between occupational exposure to pesticides and delayed commencement forms of PD in men possessing an odds ratio of 2.2 [109]. It has been reported that other specific suppressors of mitochondrial complex-I, namely rotenone (a pesticide) [110], and paraquat (a herbicide exhibiting structural resemblance with MPP+) [111], provoke deprivation of DArgic nerve cells within experimental animal models experiencing PD. 

Additionally, various epidemiological investigations have explored the association between subjection of such substances and the possibility of evolving PD. This eventually spurred the scrutiny of substitutional indicators, namely the relationship between agriculture, residing in rural regions, fertilizers [112], and consuming well water with the susceptibility of evolving PD. Subjection to welding and heavy metals comprising copper (Cu), zinc (Zn), iron (Fe), aluminum (Al), and lead (Pb), have likewise been examined, but the association between these factors and PD is still ambiguous [108].

## 5. Pathogenesis of PD

**Figure 3:**
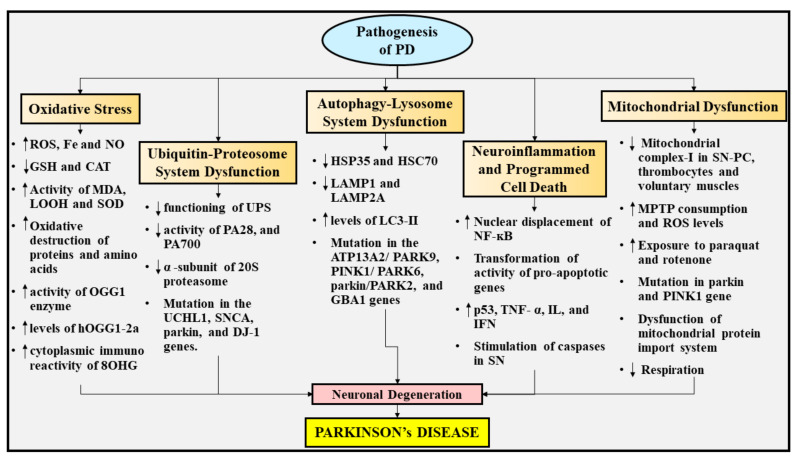
Pathogenesis of Parkinson’s disease. PD, Parkinson’s disease; ROS, reactive oxygen species; Fe, iron; NO, nitric oxide; GSH, glutathione; CAT, catalase; MDA, malondialdehyde; LOOH, lipid hydroperoxides; SOD, superoxide dismutase; OGG1, 8-oxoguanine DNA glycosylase; hOGG1-2a, hOGG1 type 2a; 8OHG, 8-hydroxyguanosine; UPS, ubiquitin-proteasome system; PA28, proteasome activator 28; PA700, proteasome activator 700; *UCHL1*, *ubiquitin C-terminal hydrolase L1*; *SNCA*, *α-synuclein*; *Parkin*, *Parkin RBR E3 ubiquitin-protein ligase*; *DJ-1*, *protein deglycase*; HSP35, hereditary spastic paraplegia 35; HSC70, heat shock cognate protein 70; LAMP1, lysosomal-associated membrane protein 1; LAMP2A, lysosomal-associated membrane protein 2A; LC3, microtubule-associated protein 1A/1B-light chain 3; *PINK1*, *PTEN-induced kinase 1*; NF-κB, nuclear factor kappa B; TNF-α, tumor necrosis factor-α; IL, interleukins; IFN, interferons; SN, substantia nigra; SN-PC, substantia nigra pars compacta; MPTP, 1-methyl-4-phenyl-1,2,3,6-tetrahydropyridine; ↑, increasing/activating/enhancing; ↓, decreasing/inhibiting/reducing.

The fundamental pathways implicated in the initiation and evolution of PD are still inexplicit, but elevated oxidative stress, UPS dysfunction, autophagy-lysosome system dysfunction, neuroinflammation, programmed cell death, and mitochondrial dysfunction probably contribute to the pathogenesis of PD. The various pathways involved in the pathogenesis of PD are depicted in Figure 3.

### 5.1. Oxidative Stress

Oxidative stress has acquired the utmost emphasis amid numerous pathogenic pathways speculated to partake in the death of DArgic nerve cells in PD. This is due to the fact that DA present within the specific areas of the brain experiences enzymatic and non-enzymatic metabolic processes in order to produce reactive oxygen species (ROS) in the SN pinpointed in the midbrain [58,113]. Elevated levels of Fe, a decline in the levels of an antioxidant named glutathione (GSH), raised quantities of a membrane polyunsaturated FA peroxidation end product termed malondialdehyde (MDA), and oxidative destruction of a long chain of amino acids and lipids have been revealed in postmortem examinations of the brains of patients experiencing PD, suggesting the significant involvement of oxidative stress in the evolution of the disease [114,115,116]. 

Likewise, the raised activity of the DNA repair enzyme termed 8-oxoguanine DNA glycosylase (OGG1) within the SN of patients experiencing PD clearly demonstrates elevated oxidation of DNA in the disease. Moreover, the levels of a subtype of human 8-oxoG DNA glycosylase (hOGG1) termed hOGG1 type 2a (hOGG1-2a) were found to be enhanced in SN of patients experiencing PD, presumably induced by mitochondrial oxidative damage [117]. Additionally, enhancement in the cytoplasmic immunoreactivity of a usual nucleic acid oxidation compound named 8-hydroxyguanosine (8OHG) has been reported in the SN of patients experiencing PD, in comparison to controlled age-matched individuals [118]. Apart from this, several other investigations have demonstrated an elevation in the lipid hydroperoxides (LOOH), functioning of superoxide dismutase (SOD), and reduction in the functioning of catalase (CAT) in patients experiencing PD. According to these researchers, MDA can indeed be the biosignature for PD, whereas LOOH and SOD are linked to delayed PD manifestations [119].

Furthermore, NO, a neurotransmitter that is produced through an amino acid, namely L-arginine, has been recognized to perform a distinctive physiological function [120]. Several investigations have revealed that NO exhibits a key function as a neurotoxic mediator related to mitochondrial impairment in a wide range of incapacitating neurodegenerative conditions, including PD [119]. In the case of diseased states, the expression of inducible nitric oxide synthase (iNOS), and nicotinamide adenine dinucleotide phosphate (NADPH) oxidase operation originates inside the microglia, culminating in extortionate synthesis of free radicals, namely NO and oxygen free radicals (O_2_^−^). The interaction between NO and O_2_^−^ prompts the generation of extremely reactive molecules, namely peroxynitrite radicals (ONOO^−^), which might precipitate the destruction of DArgic nerve cells. Apart from this, nitration of tyrosine residues emerges as a well-renowned hallmark of oxidative stress in PD patients and is actually prompted by ONOO^−^, demonstrating the significant contribution of NO generation and its secondary products in the pathogenesis of PD [121,122]. Another study has displayed that aldose reductase (AR) scarcity, a tyrosine hydroxylase (TH) cofactor actively engaged in the generation of DA, may elicit oxidative stress in animal models experiencing PD via elevating NO and nitrite (NO_2_^−^), culminating in forfeiture of DArgic nerve cells and aberrations in the autophagy-lysosome system [123]. In contrast, it has been recognized that diminished blood serum NO metabolic products, namely nitrates (NOx), and NO_2_^−^ are strongly related with cognitive dysfunction in patients experiencing PD, propounding NOx as an indicator of the early commencement form of PD [124]. Additionally, astrocytes exhibit elevated amounts of a heme-comprising peroxidase, namely myeloperoxidase (MPO), which in turn give rise to oxidative destruction via generating hypochlorous acid (HOCl) following the chemical reaction of H_2_O_2_ and chloride ions (Cl^−^) [121]. Owing to the reason that HOCl may also react with O_2_^−^, the existence of HOCl could raise the quantity of hydroxyl free radicals (OH^.^). It has been elucidated that MPO also evokes the transformation of non-reactive NO_2_^−^ into its reactive free radical state (NO_2_^−^), leading to escalation in protein destruction [125]. These findings propose the consequential participation of NO generation, iNOS activation, and the ONOO^−^ generation in nigrostriatal DArgic nerve cell degeneration, thus contributing to the pathogenesis of PD.

### 5.2. UPS Dysfunction

Various neurodegenerative diseases, which are marked by aberrant build-up of protein, comprise dysfunction in the proteasomal system as a prevalent hallmark [126]. The strongest proof of certain anomalies in PD emerges from postmortem investigations into the SN-PC, where the UPS enzymatic functioning has been reported to be considerably diminished in comparison to normal brains [127]. Afterwards, identical outcomes were observed in peripheral blood mononuclear cells (PBMCs) of patients suffering from PD, but no such effects were noticed in unaffected people [128]. In addition to declined functioning, the SN-PC of patients with PD has been reported to be associated with reduced activity of varied constituents of the proteasomal system. In particular, components which are implicated in the efficient operation of UPS, such as activators of the proteasomal system, namely proteasome activator 28 (PA28) and proteasome activator 700 (PA700) [129], and the α-subunit of the 20S proteasome are decreased [130]. Mutations in the *UCHL1*, *SNCA*, *Parkin*, and *DJ-1* gene provide insight into the proteasomal abnormality in the evolution of PD [131]. It has been elucidated that the intra striatal administration of a specific proteasome suppressor named lactacystin provokes elevation in the heme oxygenase-1 concentrations, deposition of α-synuclein, and retrograde deterioration of nerve cells within the SN, demonstrating the participation of UPS impairment in PD [132].

### 5.3. Autophagy-Lysosome System Dysfunction

There are multifarious autophagy and lysosomal concomitant constituents which have been impaired/abnormally displayed in PD, in correspondence with outcomes in the UPS pathway. Upon postmortem investigation, various molecular chaperones pertaining to the heat-shock protein class, namely hereditary spastic paraplegia 35 (HSP35) and heat shock cognate protein 70 (HSC70), as well as extremely important lysosomal membrane proteins, such as lysosomal-associated membrane protein 1 (LAMP1) and lysosomal-associated membrane protein 2A (LAMP2A), were revealed to be diminished [133,134]. It has been demonstrated that an autophagosome indicator, namely microtubule-associated protein 1A/1B-light chain 3 (LC3)-II is elevated in the SN region of the brain in patients suffering from PD, demonstrating the build-up of autophagic vesicles [135,136]. In addition, mutations in the *ATP13A2*/*PARK9* gene are thought to be strongly related to Kufor-Rakeb syndrome (KRS), an autosomal recessive kind of parkinsonian syndrome [137]. Also, it has been proven that mutations in a few additional PARK genes alter the operation of *PINK1*/*PARK6*[138] or *Parkin*/*PARK2*[139], which are both implicated in mitochondrial autophagy [140]. Furthermore, the emanation of mutations in the *GBA1* gene, which precipitates the autophagy-lysosome system impairment, as a tremendous genetic hazard for PD, lends credence to the assumption regarding the substantial involvement of this system in the evolution of PD [141]. These findings indicate that abnormal functioning of the autophagy-lysosome system partakes in PD pathophysiology.

### 5.4. Neuroinflammation and Programmed Cell Death

Neuroinflammation and programmed cell death have been reported to actively participate in PD pathophysiology. Several investigations have shown that the postmortem studies on the brains of patients suffering from PD have been identified to possess programmed cell death and autophagy [142]. Within the DArgic nerve cells of patients suffering from PD, enhancement in the nuclear displacement of NF-κB was also spotted [143]. Inflammatory processes and programmed cell death in the PD brain are further supported by transformation of activity of pro-apoptotic genes, raised concentrations of a tumor suppressor protein termed p53, NF-κB, interferon gamma (IFNγ), and stimulation of caspases inside the SN region of individuals with PD [144,145,146,147]. Activation of microglia has been portrayed within the SN region of individuals experiencing PD, whereby they result in the emission of programmed cell death-prompting inflammatory mediators such as tumor necrosis factor-α (TNF-α), interleukins (IL), and interferons (IFN) [58]. Correspondingly, stimulation of microglia is also triggered by the build-up of α-synuclein, leading to prolonged and gradual degeneration of nerve cells in the SN of PD patients [148]. Although the pathways underlying microgliosis in PD are obscure, a catecholamine-reliant dark polymer pigment, namely neuromelanin (NM)-comprising DA nerve cells, has been displayed to be incredibly prone to inflammatory processes in the disease. Moreover, it remains questionable whether inflammatory processes in the neuronal region are the chief factor for inducing PD or merely an outcome of the condition.

### 5.5. Mitochondrial Dysfunction

The impairment in mitochondrial function is presumed to be actively engaged in the pathophysiology of PD having a genetic cause or an unknown cause [149]. It has been elucidated from the early postmortem findings that a fundamental constituent of the electron transport chain (ETC), namely mitochondrial complex-I, was found to be deprived within the SN-PC region of the brain of patients suffering from PD. These findings were probably the earliest ones highlighting the direct association between mitochondrial abnormalities and PD [150]. Apart from this, deprivation in mitochondrial complex-I was also detected in the thrombocytes and voluntary muscles of individuals suffering from PD in contrast to unaffected individuals [151,152]. 

Additionally, it has been found that uncontrolled consumption of MPTP precipitates the irreversible manifestations of PD [109], along with destruction of DArgic nerve cells revealed by postmortem analysis [153]. Several studies have disclosed that DA nerve cells recognize MPTP following its oxidation into a toxic metabolite termed MPP+, which then results in the suppression of mitochondrial complex-I [154]. Moreover, paraquat (a herbicide exhibiting a structural resemblance with MPP+), and rotenone (a pesticide) are two additional toxic substances that impede the operation of the mitochondrial complex-I, resulting in the emergence of manifestations of PD and DA cell destruction plausibly in human beings and animals [77,110,111]. Therefore, mitochondrial complex-I abnormality might partake in the destruction of DA cells owing to de-escalation in the levels of energy [149]. Furthermore, mutations in the *Parkin* and *PINK1* genes provoke mitochondrial dysfunction, thereby eliciting an autosomal recessive form of PD [95,139]. 

In addition, it has been reported that α-synuclein following the binding with the membrane of mitochondria and deposition within the organelles deteriorates the operation of mitochondrial complex-I, which eventually contributes to escalated oxidative damage and mitochondrial abnormalities [155,156]. Moreover, the linkage between α-synuclein and the translocase of the inner mitochondrial membrane 20 (TOM20) evokes abnormality in the import system of the mitochondrial protein, profuse synthesis of ROS, and a decline in breathing [157]. These factors share their significant contribution to mitochondrial dysfunction.

## 6. Experimental Studies Portraying the Deep Insights into the Neuroprotective Role of PPAR Agonists in PD 

It has been elucidated that DArgic nerve cell degeneration is spurred by the generation of ROS, which in turn induces oxidative destruction, microglia-effectuated inflammation in the neuronal region, and mitochondrial abnormalities, and each of these in conjunction contributes to the stimulation of programmed cell death. Consequently, modulation of oxidative stress and mitochondrial abnormalities might assist in restraining the decline in functioning of nerve cells in PD [31,58]. In accordance with numerous investigations, it has been revealed that PPAR agonists exhibit neuroprotective actions in various in vivo and in vitro models experiencing PD.

### 6.1. Therapeutic Implications of PPAR-γ Agonists in PD 

It has been reported that following the oral delivery of the PPAR-

γ agonist, namely pioglitazone (20 mg/kg) before *i.p* administration of MPTP (in a dose of 15 mg/kg) resulted in a reduction in MPTP-inebriation prompted microglia stimulation and precluded forfeiture of DArgic nerve cells within the SN-PC of an experimental model of mouse experiencing PD [158]. Furthermore, another investigation has revealed the safeguarding outcomes of pioglitazone in the case of MPTP-instigated neurotoxicity, which agrees with the results of previous investigations [159]. The safeguarding action of pioglitazone in the case of MPTP-prompted neurotoxicity is exerted via the inhibition of the transformation of MPTP to its deleterious metabolic product, MPP+, through monoamine oxidase B (MAOB) suppression [160,161]. It has been proven that after oral delivery of pioglitazone, significant shielding was extended towards MPTP-prompted nerve cell destruction in TH-immunoreactive SN nerve cells [159]. 

Therapy with the aid of pioglitazone gives rise to substantially decreased stimulation of microglia, nitro tyrosine activity in DArgic nerve cells, mediators of inflammatory processes, and the fraction of glial fibrillary acidic protein (GFAP) positive cells in the SN region and striate nucleus [159]. Several studies have reported that PPAR-γ agonists possess anti-inflammatory [15,17,162], and anti-neoplastic properties [15,17]. In experimental studies, oral delivery of pioglitazone rapidly following the emergence of PD in rhesus monkeys resulted in DA striatal fibers and SN nerve cells preservation, thereby exhibiting a neuroprotective role in PD [163]. Another investigation has shown that the oral introduction of pioglitazone (in a dose of 30 mg/kg) following 6-hydroxyDA(6-OHDA) lesions in male wistar rats experiencing PD resulted in rendering protection to DArgic nerve cells in the SN against neuronal destruction and offered a significant decrease in NF-κB and microglial stimulation [164]. A recent study has elaborated on the neuroprotective outcomes of pioglitazone in both in vivo and in vitro MPTP or MPP+ provoked PD models. 

In vivo investigation has demonstrated that oral delivery of pioglitazone led to considerable improvement in behavioral manifestations destructed by MPTP, as well as the escalation in the lifespan of TH positive nerve cells, a fraction of cell powerhouses and improved mitochondrial ultrastructure, and enhanced activity of PGC-1α. In vitro investigation has revealed the modulation of activity of numerous proteins implicated in the operation of mitochondria, for instance, PGC-1α, mitochondrial fission 1 (Fis1), mitochondria fusion 2 (MFN2), nuclear respiratory factor 1 (NRF1), and nuclear respiratory factor 2 (NRF2) after oral treatment with pioglitazone [165]. 

Additionally, it has been stated that pioglitazone renders neuroprotection in intra-striatally delivered lipopolysaccharide (LPS) triggered inflammatory processes in an experimental model of rat possessing degeneration of DArgic nerve cells [166,167]. According to this investigation, treatment with the assistance of pioglitazone considerably evoked decline in LPS triggered microglial inflammatory processes, oxidative damage, upgraded the operation of mitochondria, incompletely reinstated DA concentrations, and enhanced DArgic neuroprotection. 

Furthermore, a related study highlighted the neuroprotective pathway exhibited by pioglitazone towards LPS [168]. Through meddling with two processes, namely NF-κB and Jun N-terminal kinase (JNK), which reduce the expression of COX-2, pioglitazone safeguarded DArgic nerve cells against LPS prompted COX-2 and prostaglandin E2 (PGE2) driven intensification of stimulation of microglia in DArgic nerve cells-neuroglia co-cultures [168]. Apart from this, pioglitazone also safeguards DArgic nerve cells from LPS-prompted impairment via diminishing the expression of iNOS, and synthesis of NO through distinguishably controlling the p38 mitogen-activated protein kinase (MAPK), and phosphoionositide 3-kinase (PI3K)/Akt processes [169].

Moreover, i.p. administration of another PPAR-γ agonist, namely rosiglitazone (in a dose of 10 mg/kg) in the MPTP or probenecid (MPTPp) animal model of mice (C57BL/6J) experiencing PD resulted in a decline in the generation of inflammatory mediator, namely TNF-α in the microglia cells, and arrested MPTPp-prompted degeneration of nerve cells in the SN-PC [170]. Recently, it has been elaborated that rosiglitazone holds the aptitude to safeguard retinal nerve cells from the abnormalities precipitated by exposure to rotenone and escalates neuroprotection in the retina, and CNS of rotenone-prompted rat models of PD, following the systemic delivery of rosiglitazone in liposome-encapsulated form (1 mL/kg, i.p.) [171]. 

Furthermore, it has been discovered that rosiglitazone safeguards human neuroblastoma cells from a mitochondrial operation suppressor and neurotoxin, namely acetaldehyde (CH_3_CHO), and a neurotoxin, namely MPP+ [172,173]. Acetaldehyde elicits nerve cell destruction via promoting programmed cell death and intracellular release of ROS [172]. Through spurring the activity of antioxidant enzymes and controlling the expression of programmed cell death regulators, namely Bax and Bcl-2, rosiglitazone was capable of repressing acetaldehyde-prompted programmed cell death [172]. Rosiglitazone safeguards human neuroblastoma cells from MPP+ prompted cellular damage through the suppression of abnormalities in the functioning of mitochondria and ROS generation [173]. These findings indicate that apart from the anti-inflammatory property of PPAR agonists, they also safeguard nerve cells by modulating the activity of CAT and SOD (antioxidant enzymes) and maintaining an equilibrium between the activity of pro-apoptotic genes and anti-apoptotic genes [172,173]. 

### 6.2. Therapeutic Implications of PPAR-β/δ Agonists in PD

It has been demonstrated that in the rotenone-prompted rat model of PD, a PPAR-β/δ agonist, namely GW-501516, safeguards DArgic nerve cells from destruction provoked by deleterious substances, as well as upgrades behavioral performance by diminishing ER-related stress in PD. Moreover, in the MPTP instigated PD model of mice, a decline in nucleotide-binding domain and leucine-rich-repeat-protein 3 (NLRP3) inflammasome-related nerve cell inflammation is rendered by PPAR-β/δ agonists [174]. 

Another study revealed that PPAR-β/δ agonists, namely GW-501516 and L-165041, remarkably safeguarded SH-SY5Y neuroblastoma cells from staurosporine and MPP+ triggered programmed cell death primarily through suppressing the stimulation of the caspase-3 pathway [10]. Additionally, these agonists safeguarded against MPTP-instigated DA and its metabolic products deprivation in the SN-PC [10]. Besides, a recent investigation has revealed the outcomes of 2-[4-[[2-[3-fluoro-4-(trifluoromethyl) phenyl]-4-methyl-1,3-thiazol-5-yl] methyl sulfanyl-[2-methylphenoxy] acetic acid (GW0742), a PPAR-β/δ agonist in an experimental model of rat experiencing PD-related cognitive abnormalities. In accordance with this investigation, MPTP exposure to rats led to oxidative destruction and splitting-up of DNA strands into fragments. Thereafter, therapy with the aid of GW0742 (in a dose of 30 and 100 µg/kg) resulted in incomplete reinstatement of MPTP-damaged cognitive activities. Furthermore, it has been shown that GW0742 significantly minimized oxidative destruction and splitting-up of DNA strands into fragments in numerous assays, including immunochemical (Tunel), MDA, and GSH [175,176]. 

### 6.3. Therapeutic Implications of PPAR-α, and PPAR-α/γ Agonists in PD 

In addition to PPAR-γ, and PPAR-β/δ agonists, several PPAR-α, and PPAR-α/γ agonists have been shown to exert a neuroprotective action in different PD models. Fenofibrate and benzafibrate are the two PPAR-α agonists that have been extensively examined in the MPTP-treated experimental mouse model of PD [177]. One study has revealed that fenofibrate potentially safeguarded DArgic nerve cells in the SN and TH-immunoreactive endings within the striatal region, however benzafibrate displayed no such safeguarding action, this can possibly be accounted by the fact that benzafibrate was administered at a 10 times lesser amount in comparison to fenofibrate [177]. 

Another study has reported that oral delivery of fenofibrate (in a dose of 100 mg/kg) in an experimental rat model of PD following one hour of MPTP exposure contributed to a significant decline in hypo-locomotion precipitated by MPTP, depressive behavioral patterns following neurotoxin administration, prevented both an escalation in ROS generation and a decline in DA concentrations following surgical procedures, thereby demonstrating neuroprotective action in the MPTP-prompted PD model [178]. These studies suggest that therapy with the assistance of fenofibrate holds the potential to impede the evolution of PD [59].

A recent study has stated that prior treatment with the aid of the PPAR-α/γ dual agonist, namely 2-[4-(5-chloro-1,3-benzothiazol-2-yl)phenoxy]-2-methylpropanoic acid (MHY908), had demonstrated neuroprotective action against MPTP-prompted experimental mouse models of PD [179]. The neuroprotection in this PD model was rendered via reduction in MPTP-prompted DArgic nerve cell deprivation and motor impairment, alleviation of MPTP-prompted stimulation of glial cells in the nigrostriatal region, suppression of MPP+ prompted stimulation of astroglia via inhibition of NF-κB signaling in primary cultured astrocytes, and suppression of MPP+ prompted cell destruction and ROS generation in SH-SY5Y neuroblastoma cells [179]. 

**Figure 4:**
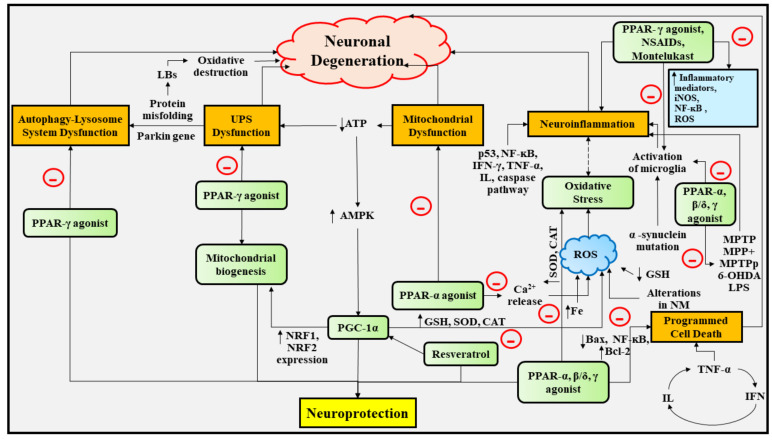
Pathways implicated in neuroprotection by means of PPARs in Parkinson’s disease. Genetic mutations as well as environmental factors play a pivotal role in the evolution of PD. Oxidative stress, UPS dysfunction, autophagy-lysosome system dysfunction, mitochondrial dysfunction, neuroinflammation, and programmed cell death contribute to the pathogenesis of PD. Each of these contributing factors may not be present simultaneously, and their association appears to participate in PD pathogenesis. Exposure to environmental toxins provokes oxidative stress, and activation of microglia. In addition, α-synuclein mutation also leads to the activation of microglia, and further provokes neuroinflammation. Activation of microglia in turn triggers the liberation of mediators of inflammation and ROS generation, which eventually prompt nerve cell degeneration via programmed cell death or necrosis. Analogously, apoptotic nerve cells liberate ROS and a variety of pernicious substances, which might aggravate the inflammatory processes precipitated via the activation of microglia. Ca^2+^ release, increased Fe levels, decreased GSH, and alterations in NM promote ROS generation, which leads to oxidative stress. Decrease in ATP as a result of mitochondrial dysfunction results in UPS dysfunction, aberrant protein build-up, and oxidative destruction effectuated neuronal degeneration. *Parkin* gene mutations result in autophagy-lysosome system dysfunction and mitochondrial dysfunction, which consecutively promotes neuronal degeneration. PPAR agonists render neuronal protection through the suppression of autophagy-lysosome system dysfunction, UPS dysfunction, mitochondrial dysfunction, oxidative stress, neuroinflammation, and programmed cell death. PPAR agonists, by elevating the levels of SOD and CAT decrease the oxidative stress. These agonists suppress the toxin exposure triggered activation of microglia. Further, PPAR agonists, NSAIDs, and montelukast diminish the expression of iNOS, NF-κB, ROS, inflammatory mediators, microglia activation, and neuroinflammation. Analogously, they also decrease the programmed cell death via suppressing the phosphorylation of Bax and NF-κB expression, and by prompting the expression of Bcl-2. Besides, a PPAR-γ-coactivator, namely PGC-1α decreases oxidative stress by elevating the levels of GSH, SOD, and CAT, and provokes the NRF1 and NRF2 (downstream target genes) expression implicated in mitochondrial biogenesis, thereby rendering neuronal protection. Apart from this, an antioxidant, namely resveratrol, also exhibits neuroprotective action by activating PGC-1α. Minus sign denotes suppressing/diminishing action, whereas arrow denotes promoting action. LBs, lewy bodies; *Parkin*, *Parkin RBR E3 ubiquitin-protein ligase*; UPS, ubiquitin-proteasome system; NRF1, nuclear respiratory factor 1; NRF2, nuclear respiratory factor 2; ATP, Adenosine triphosphate; AMPK, AMP-activated protein kinase; PGC-1α, PPAR-gamma co-activator-1 alpha; GSH, glutathione; SOD, superoxide dismutase; CAT, catalase; Ca^2+^, calcium; Fe, iron; NM, neuromelanin; ROS, reactive oxygen species; NF-κB, nuclear factor kappa B; IFNγ, interferon gamma; TNF-α, tumor necrosis factor-α; IL, interleukins; NSAIDs, non-steroidal anti-inflammatory drugs; iNOS, inducible nitric oxide synthase; MPTP, 1-methyl-4-phenyl-1,2,3,6-tetrahydropyridine; MPTPp, probenecid model of Parkinson’s disease; MPP+, 1-methyl-4-phenylpyridinium ion; 6-OHDA, 6-hydroxyDA; LPS, lipopolysaccharide; Bcl-2, B-cell lymphoma 2; IFN, interferons; PD, Parkinson’s disease; ↑, increasing/activating/enhancing; ↓, decreasing/inhibiting/reducing.

Table 1 presents the neuroprotective outcomes of PPAR agonists in PD and the pathways by which PPARs exhibit neuroprotective action in PD are depicted in Figure 4.

### 6.4. Therapeutic Implications of NSAIDs, Leukotriene Receptor Antagonist, and Vitamin E in PD 

Apart from this, several commonly employed NSAIDs, for instance, ibuprofen, fenoprofen, flufenamic acid, naproxen, and indomethacin explicitly interact either with PPAR-α/PPAR-γ or both and culminate in their activation [27,190,191]. Numerous researchers have explored the efficacy of ibuprofen and indomethacin in rendering significant neuroprotection in PD and other neurodegenerative diseases [51,190,192]. According to one study, in mesencephalic cultured cells, paracetamol (1mM) and ibuprofen (0.1mM) effectively mitigated 6-OHDA, MPP+, and glutamate prompted DArgic nerve cell destruction [180,181]. 

Moreover, it has been stated that ibuprofen individually raised the quantity of DArgic nerve cells by approximately 47% [180]. A recent study has reported that multimodal therapy with an iminosugar, namely 1-deoxynojirimycin (1-DNJ) and a NSAID, namely ibuprofen, impedes destruction of mesencephalic DArgic nerve cells, minimizes the levels of inflammatory mediators like interleukin-6 (IL-6) and TNF-α, total microglia markers namely CD68^+^/Iba-1^+^ cells, and interaction between microglia cells and nerve cells in MPTP-subjected experimental mice model [182]. 

Additionally, a new investigation has demonstrated that co-treatment with a novel herbal mixture comprising 12 medicinal herbs, namely Gagam-Sipjeondaebo-Tang (GST) and ibuprofen, exhibited a synergistic action in ameliorating DArgic nerve cell destruction and reducing the activation of macrophages in the MPTP-prompted mouse model of PD [183]. Further, the levels of NO were considerably declined in LPS-activated macrophages following this co-treatment. According to this investigation, GST alone remarkably reduced DArgic nerve cell death, levels of IL-6, COX-2, iNOS, and interleukin-1 beta (IL-1β), and relieved PD-related behavioral abnormalities [183]. Another study revealed that in the MPTP prompted experimental model of mice, indomethacin extended safeguardance towards MPTP-prompted nerve cell destruction and diminished activation of microglia and the infiltration of lymphocytes [184]. 

Furthermore, several other agents have been proven to exert a neuroprotective action on PD, including celecoxib (a selective COX-2 inhibitor) [185], montelukast (a leukotriene receptor antagonist) [186,187,188,193], and tocopherol (vitamin E) [194,195]. Therapy with the aid of celecoxib (< 20 μM) has been shown to reinstate SH-SY5Y cells that had been potentially subjected to paraquat and 6-OHDA prompted damage [185]. Additionally, celecoxib therapy culminated in a significant and persistent overexpression of a lipocalin carrier of tiny hydrophobic molecules, namely apolipoprotein D (APOD), as well as a few of the microphthalmia transcription factors, namely microphthalmia-associated transcription factor (MITF) and transcription factor E-box binding (TFEB). Thus, celecoxib holds the aptitude to diminish the symptoms and evolution of PD by exerting its neuroprotective action by means of safeguarding the DArgic nerve cells from damage [185]. 

In an experimental mouse model of PD, montelukast exhibited safeguardance to DA nerve cells against the activation of microglia cells and reduced the generation of IL-1β and TNF-α [186]. Another study revealed that montelukast treatment resulted in a reduction in rotenone-prompted activation of microglia cells and safeguarded motor activities from impairment [187]. A more in-depth investigation into the role of montelukast in the rotenone-prompted PD rat model indicated a decline in activation of microglia cells and an upgradation in motor activities [188]. Moreover, administration of montelukast contributed to a significant reduction in p53 protein and decreased oxidative damage owing to montelukast’s ROS scavenging ability, thereby having a strong impact on the lifespan of nerve cells [188]. Vitamin E, owing to its antioxidant activity, may possess a neuroprotective action against PD, but the underlying pathways through which it exhibits neuroprotective action remain unclear [194]. These findings suggest that these agents can contribute to neuroprotection against PD via specific mechanisms.

### 6.5. Therapeutic Implications of PGC-1α in PD 

The transcriptional coactivator, namely PGC-1α, is a fundamental modulator of mitochondrial biogenesis and operation, encompassing oxidative phosphorylation and elimination of ROS. PGC-1α is widely distributed in tissues that necessitate an enormous amount of energy [196]. The relationship between PD and variations in mitochondrial equilibrium has been observed [197]. Several investigations have been conducted in order to adequately scrutinize the involvement of PGC-1α in PD. It has been demonstrated that PGC-1α causes a significant decrease in oxidative stress via eliciting the activity of enzymes that possess ROS scavenging ability, such as glutathione peroxidase-1, SOD, and CAT [189]. PGC-1α genetically inactivated mice have displayed an elevated predisposition to MPTP-prompted degeneration of DArgic nerve cells in SN-PC, implying that PGC-1α possess remarkable neuroprotective effects. As a consequence, up-regulation of PGC-1α provoked mitochondrial biogenesis, and markedly safeguarded nerve cells from oxidative damage [189]. PGC-1α stimulation resulted in enhanced expression of nuclear-encoded ETC components as well as restrained DArgic nerve cell decline provoked by mutations in α-synuclein or exposure to rotenone in PD models [198]. Additionally, in human nerve cells, inactivation of PGC-1α raised the build-up of α-synuclein and eventually culminated in de-escalation of the Akt/GSK-3beta signaling mechanism [19,199]. The parkin-interacting substrate (PARIS), a parkin substrate, is a Zn-finger protein (ZFP) that is extensively located in the SN region. PARIS has been reported to suppress PGC-1α and NRF expression, and the connecting region between PARIS and PGC-1α is a pattern which actively participates in modulating metabolism of energy and pancreatic hormone (insulin) responsiveness. Experimental adult animals with a stipulatory inactivation of parkin experienced gradual destruction of DA nerve cells that was reliant upon the expression of PARIS. Furthermore, up-regulation in the expression of PARIS sparked specific DA nerve cell decline in the SN, which was rescued through the co-expression of *Parkin*/PGC-1α [200]. According to a new study, the mutations in the *PINK1* gene disrupt parkin recruitment to energy factories in nerve cells, elevating mitochondrial copy numbers and PGC-1α overexpression [201]. Another investigation has revealed that up-regulating PGC-1α transgenicity or activating PGC-1α with the assistance of a polyphenol, namely resveratrol (an antioxidant), safeguards DArgic nerve cells in the MPTP animal model of PD [202]. These findings highlight that PGC-1α partakes in the pathogenesis of neurodegenerative diseases, and therefore could be a promising therapeutic target for such devastating and incapacitating diseases [19,203]. However, much research is essential to adequately unravel the molecular pathways by which PGC-1α modulates PPAR transcription in the CNS. Apart from the significant neuroprotective action of PPAR agonists in PD, these agonists also provide neuroprotection in numerous neurodegenerative diseases, such as AD, HD, and ALS.

### 6.6. Therapeutic Implications of Smoking, Caffeine, and Alcohol Consumption in PD

The consequences of smoking on PD have been eminently scrutinized, with relatively identical outcomes. The preponderance of epidemiological findings are case-referent studies that indicate a diminished possibility of acquiring PD, which is further confirmed by substantially bigger cohort studies [204,205,206]. An enormous meta-analyses comprising 8 cohort studies and 44 case-referent studies across twenty countries discovered an inversely proportional relationship between cigarette smoking and PD, with a cumulative hazard of 0.39 for active cigarette smokers [207]. Additionally, a few meta-analyses also revealed an inversely proportional relationship between cigarette smoking and PD, with a cumulative odds value varying between 0.23–0.70, implying a safeguarding approach towards PD [208,209].

Furthermore, researchers have also discovered an inversely proportional relationship between the total count of pack years, years of cigarette smoking, and the potential hazard of PD, with perennial or chronic cigarette smokers possessing a considerably decreased susceptibility to instigating PD in comparison to those who do not smoke [208]. There are numerous explanations suggesting the protective action of cigarette smoking on the susceptibility to developing PD, but they are still poorly understood [210,211]. Nicotine, a chiral alkaloid, which triggers the stimulation of DArgic nerve cells, alleviation of manifestations associated with PD, and also possesses a neuroprotective outcome, has spurred the most interest among the various chemical constituents present in cigarette smoke [211].

The influence of five distinct chemical constituents of cigarette smoke, namely anabasine, nicotine, hydroquinone, nomicotine, and cotinine upon the fibrillation of a protein named α-synuclein (which accumulates in LBs, and several other proteins in the case of PD), was explored in a recent investigation. It has been reported that nicotine and hydroquinone suppress the production of α-synuclein fibrils, with nicotine emerging as the more potent suppressor, implying that both the chemical constituents maintain soluble oligomeric forms of the protein [212]. However, nicotine can also induce DA release, which is implicated in reward processes, rendering it abstruse and perplexing to determine whether cigarette smoking aids in the prevention of PD or PD assists people to stop smoking. Patients experiencing PD might be less susceptible to compulsive actions, and therefore less probable to smoke cigarettes because of a decline in DA levels. This explanation is strengthened by the evidence that patients suffering from prefatory PD and PD hold the capability to cease cigarette smoking considerably more readily in comparison to controls, indicating that the diminished reactivity to nicotine may be liable for this correlation [213].

Numerous researchers have explored the action of caffeine (a most extensively utilized psychoactive agent) intake on the evolution of PD and discovered that individuals consuming coffee are less prone to the condition [214,215,216]. Caffeine belongs to the class of purinergic P1 adenosine (ADO) A_2A_ receptor inhibitors, which are considered to exert a beneficial action on patients experiencing PD [217], and has been proven to exhibit a neuroprotective role in experimental mouse models experiencing PD [218]. Individuals consuming coffee possess a lower incidence of evolving PD, with a respective incidence varying from 0.45–0.80 in coffee consumers in comparison to individuals not consuming coffee, as per two large prospective epidemiological investigations [217,219], and numerous case-referent studies [220]. Moreover, according to a meta-analysis that comprised five cohort studies and eight case-referent studies, there is a substantially lower incidence of evolving PD (with a risk ratio of 0.69) in individuals consuming coffee [207]. Apart from this, consumption of tea has also been associated with a decreased incidence of evolving PD [216]. As with smoking, he mechanism by which caffeine exhibits protective action against PD is yet unknown. In addition, gender variations have been observed in several investigations. It has been reported that in 2 cohort studies, coffee has displayed a slightly elevated inversely proportional relationship in the evolution of PD in males as compared to females [217,219]. Moreover, the action of caffeine in post-menopausal women was reliant upon whether the women were or were not on estrogen-containing hormone replacement therapy (HRT). Because estrogen suppresses the metabolic processes that carry out degradation of caffeine, the interplay between estrogen and caffeine might elucidate the reason why/how HRT influences the incidence of PD in post-menopausal women [221].

According to a recent investigation and meta-analysis of case-referent studies, an inversely proportional relationship has been discovered amongst intake of alcohol and the vulnerability of evolving PD, while considering both chronic/modest consumption of alcohol with no/slight intake of alcohol, and “never” versus “ever” ingestion of alcohol [222]. This meta-analysis comprised 26 suitable retrospective case-referent studies, and five prospective longitudinal cohort studies on ingestion of alcohol and PD involving 8798 patients experiencing PD and15,699 control subjects, and 2404 patients experiencing PD and 600,592 control subjects, respectively. Retrospective studies have reported that following the comparison between patients experiencing PD and control subjects, the proportion of never drinkers was considerably greater than the proportion of chronic or/and modest drinkers (diagnostic odds ratios (95% confidence intervals) 1.33 (1.20–1.48), and 0.74 (0.64–0.85)), sequentially [222]. Contrastingly, prospective studies have revealed insignificant variations apart from a shift toward a substantially elevated prevalence of non-alcohol consumers in PD females than modest or/and chronic alcohol consumers in PD males among those investigations that distinguished results on the basis of gender [222]. This meta-analysis strongly demonstrated an inversely proportional relationship between alcohol intake and evolution of PD, which is corroborated by case-referent studies, but however not by prospective studies. Another meta-analysis of non-experimental studies examined the relationship between consumption of alcohol and evolution of PD. In accordance with this meta-analysis involving 32 studies, and comprising 677,550 patients, it has been elucidated that beer (risk ratio= 0.59, 95% confidence intervals: 0.39–0.90), but not wine or liquor, potentially safeguarded against the emergence of PD, particularly for men (risk ratio= 0.65, 95% confidence intervals: 0.47–0.90), although this did not extend to women [223,224]. However, there have been insufficient investigations performed on dose-response assessment and the interactions among beer, wine, and liquor. Owing to this obscurity, the outcomes of these investigations are contradictory. The association between consumption of alcohol and emergence of PD is intricate, and further research is essential in order to achieve evidenced based outcomes.

### 6.7. Therapeutic Implications of Physical Exercise in PD

It has been elucidated that physical exercise escalates mitochondrial energy generation, decreases inflammatory processes, triggers new blood vessel formation (angiogenesis), promotes antioxidant safeguardance, and elicits the synapse formation between nerve cells in the nervous system (synaptogenesis), and thereby could be a plausible strategy for the advancement of nerve cell protective and restorative therapy for PD [225]. Performing physical exercise is also accordant with a growing body of corroboration demonstrating the benefits of physical treatment in addressing motor deficits and upgrading cognitive and emotional well-being. The credence that physical exercise might assist in hindering the commencement and evolution of PD has incentivized healthcare practitioners to strongly suggest physical exercise to their patients. Furthermore, a growing body of corroboration reveals that physical exercise does assist patients experiencing PD in the same way as it assists people with other neurological conditions, for instance AD, HD, ALS, spinal column injury, and stroke [225]. It has been reported that switching to the utilization of running in a wheel or on a treadmill has gathered a great amount of corroboration that these kinds of physical exercises significantly de-escalate the behavioral repercussions of MPTP or 6-OHDA exposure in experimental models of monkey, rat, and mouse [225]. This is backed by several other investigations in the experimental rat model subjected to 6-OHDA [226,227,228,229], and an experimental mouse model subjected to MPTP [230]. Furthermore, mice of two to four months in age were provided with accessibility to a running wheel directly connected with their cages (for a duration of three months), the animals being sacrificed following one or two weeks of MPTP (in a dose of 4x20 mg/kg at 120 min intervals, i.p.) introduction; the quantity of TH immunoreactive cells in the SN region was assessed employing stereology. MPTP-subjected animals lacking accessibility to a running wheel had revealed a 42% decline in TH immunoreactive cells, while those exhibiting accessibility to running wheels had revealed just a 9% decline in TH immunoreactive cells [225]. Another investigation looked at adult male rats provided constant accessibility to a running wheel for a duration of 12 weeks, after which 6-OHDA was subjected into the nigrostriatal projection (in a dose of 0.6mg in 2 mL) and were then brought back to the same cage (rendering constant accessibility to a running wheel) for the next eight weeks, whereas control animals were not able to access the running wheel. 6-OHDA subjected rats in cages being inaccessible to running wheels dropped nearly 47% of TH immunoreactive cells, and 49% of TH immunoreactive cells in the striatal region, which was further escorted by a 36% decline in DA levels in the striatal region, each of these considerably decreased by means of physical exercise [225]. The relationship between physical exercise and PD emergence, and neuroprotective outcomes of physical exercise remains to be perplexed and equivocal, and additional research is required to attain novel propitious treatments for the therapy of PD.

## 7. Conclusions

Existing pharmacotherapy can palliate the manifestations associated with PD, but no therapy has been displayed to eradicate the disease evolution. Consequently, neurotherapeutic substances that can render neuronal protection are critically desired in order to treat this intricate and incapacitating condition. PD, being a multifaceted condition, emerges due to the contribution of genetic mutations and environmental factors. The molecular mechanisms, encompassing oxidative stress, UPS dysfunction, autophagy-lysosome system dysfunction, neuroinflammation and programmed cell death, and mitochondrial dysfunction are actively engaged in the pathogenesis of PD. Due to the single target regulation offered by existing pharmacotherapy, this makes the abolition of disease progression all but impossible. Thus, neuronal protection can be effectively accomplished with the aid of such pharmacological substances that hold the potential to regulate multiple molecular and pathogenic mechanisms at the same time. New expanding corroborations have revealed that PPAR agonists have the potential to alter and regulate multiple molecular mechanisms at the transcriptional level via prompting gene expression. Numerous PPAR agonists/substances, for instance, pioglitazone, rosiglitazone, GW-501516, L-165041, GW0742, fenofibrate, benzafibrate, and MHY908 have been reported to act at the transcriptional level and thereby emerge as neoteric and propitious targets in the therapy of neurodegenerative diseases. Further experimental studies are needed to gain an in-depth understanding of PPARs, their agonists, their neuroprotective outcomes, and their benefits and shortcomings in order to overcome neuronal degeneration. Eventually, a comprehensive knowledge of the molecular pathways through which PPARs render neuronal protection would assist in the development of a potentially effective treatment into clinical practice for the therapy of PD.

## Figures and Tables

**Table 1:** The neuroprotective outcomes of PPAR agonists in Parkinson’s disease.

PPAR Agent/ Ligand	Compound/ Toxin Utilized	Animal Model/ Cell Type	Outcomes	Ref.
1. PPAR-γ agonist				
Pioglitazone (oral, 20 mg/kg)	MPTP (i.p, 15 mg/kg)	Mouse	Reduced MPTP-inebriation prompted microglia stimulation. Precluded forfeiture of DArgic nerve cells within the SN-PC.	[158]
Pioglitazone (oral)	MPTP	Mouse	Extended safeguardance towards MPTP-prompted nerve cell destruction in TH-immunoreactive SN nerve cells. Decreased stimulation of microglia, inflammatory mediators, nitro tyrosine activity in DArgic nerve cells, and the fraction of GFAP positive cells in the SN and striate nucleus.	[159]
	MPTP	Rhesus monkeys	Preserved DA striatal fibers and SN nerve cells.	[163]
Pioglitazone (oral, 30 mg/kg)	6-OHDA	Male Wistar Rats	Rendered protection to DArgic nerve cells in the SN against neuronal destruction. Offered a significant reduction in NF-κB and microglial stimulation.	[164]
Pioglitazone	LPS (i.c.v)	Rat	Decreased LPS triggered microglial inflammatory processes, and oxidative damage. Upgraded mitochondrial operation, incompletely reinstated DA levels, and enhanced DArgic neuroprotection.	[166,167]
	LPS	DArgic nerve cells-neuroglia co-culture	Safeguarded DA nerve cells via suppression of microglial activation, diminished phosphorylation of NF-κB and JNK, and reduced expression of COX-2. Diminished expression of iNOS, and synthesis of NO through distinguishably modulating the p38 MAPK and PI3K/Akt processes.	[168,169]
Rosiglitazone (i.p, 10 mg/kg)	MPTP/MPTPp	Mice (C57BL/6J)	Decreased generation of TNF-α in the microglia cells, and arrested MPTPp-instigated nerve cell degeneration in the SN-PC.	[170]
Rosiglitazone (liposome-encapsulated form), (i.p, 1mL/kg)	Rotenone	Rat	Can safeguard retinal nerve cells from the abnormalities provoked by subjection to rotenone, and elevated neuroprotection in the retina and CNS.	[171]
Rosiglitazone	Acetaldehyde	Human neuroblastoma SH-SY5Y cells	Safeguarded DA nerve cells from acetaldehyde prompted programmed cell death via enhancing the activity of antioxidant enzymes, and by controlling the expression of Bax and Bcl-2.	[172]
	MPP+		Safeguards SH-SY5Y cells from MPP+ prompted cellular damage via the suppression of impairment in the functioning of mitochondria and ROS generation. Raised CAT, SOD, Bcl-2 expression, and diminished Bax expression.	[173]
2. PPAR-β/δ agonist				
GW-501516	Rotenone	Rat	Safeguards DArgic nerve cells from damage caused by deleterious substances and upgrades behavioral performance by diminishing ER-related stress.	[174]
GW-501516 and L-165041	Staurosporine, and MPP+	SH-SY5Y cells	Safeguarded SH-SY5Y cells from staurosporine and MPP+ elicited programmed cell death via suppressing the caspase-3 pathway activation.	[10]
GW0742 (30 and 100 μg/kg)	MPTP	Rat	Incomplete reinstatement of MPTP-damaged cognitive activities. Reduced oxidative destruction and splitting-up of DNA strands into fragments.	[175,176]
3. PPAR-α agonist				
Fenofibrate (0.2% in diet) and Benzafibrate (0.02% in diet)	MPTP	Mouse	Fenofibrate safeguarded DArgic nerve cells in the SN and TH-immunoreactive endings within the striatal region, however benzafibrate displayed no such safeguarding action.	[177]
Fenofibrate (oral, 100 mg/kg)		Rat	Reduced MPTP provoked hypo locomotion, and depressive behavioral patterns after neurotoxin administration. Safeguarded from elevation in the ROS generation and decrease in levels of DA following surgical procedure.	[178]
4. PPAR-α/γ dual agonist				
MHY908	MPTP	Mouse	Reduced MPTP-prompted DArgic nerve cell deprivation, and motor impairment. Alleviation of MPTP-instigated activation of glial cells in the nigrostriatal region. Suppression of MPP+ prompted activation of astroglia by inhibition of NF-signaling in primary cultured astrocytes. Suppression of MPP+ provoked cellular damage and ROS generation in SH-SY5Y neuroblastoma cells.	[179]
5. NSAIDs				
Paracetamol (1 mM), and Ibuprofen (0.1 mM)	6-OHDA, MPP+, and glutamate	Mesencephalic cultured cells	Effectively mitigated 6-OHDA, MPP+, and glutamate prompted DArgic nerve cell death. Ibuprofen individually elevated the quantity of DArgic nerve cells by nearly 47%.	[180,181]
1-DNJ + Ibuprofen	MPTP	Mice	Impedes mesencephalic DArgic nerve cell death. Minimizes the levels of IL-6, TNF-α, total microglia markers namely CD68^+^/Iba-1^+^ cells, and interaction between microglia cells and nerve cells.	[182]
GST + Ibuprofen		Mouse	Exhibited a synergistic action in ameliorating DArgic nerve cell death and reducing the activation of macrophages. Reduced NO levels in LPS-activated macrophages. GST alone reduced DArgic nerve cell death, levels of iNOS, IL-6, IL-1β, and COX-2, and relieved PD-concerned behavioral impairment.	[183]
Indomethacin		Mice	Extended safeguard towards MPTP-prompted nerve cell destruction. Diminished infiltration of lymphocytes, and microglia activation.	[184]
Celecoxib (< 20 uM)	Paraquat, and 6-OHDA	SH-SY5Y cells	Reinstated SH-SY5Y cells from damage caused by exposure to paraquat and 6-OHDA. Resulted in prolonged overexpression of APOD, MITF, and TFEB, and safeguarded DArgic nerve cell from damage.	[185]
6. Leukotriene receptor antagonist				
Montelukast	6-OHDA	Mouse	Safeguarded DA nerve cells against microglia cells activation, and reduced the generation of IL-1β and TNF-α.	[186]
Montelukast	Rotenone	Rat	Reduced microglia cells activation and upgraded motor activities. Decreased p53 protein, oxidative damage, thereby strongly influences life span of nerve cells.	[187,188]
7. PGC-1α				
PGC-1α	MPTP	PGC-1α genetically inactivated mice	Elevated proneness to MPTP prompted degeneration of DArgic nerve cells in SN-PC. Up-regulation of PGC-1α provoked mitochondrial biogenesis, and safeguarded nerve cells from oxidative damage.	[189]

Legend: PPAR, Peroxisome proliferator-activated receptor; GW-501516, 2-[2-methyl-4-[[4-methyl-2-[4-(trifluoromethyl)phenyl]-1,3-thiazol-5-yl]methylsulfonyl]phenoxy]acetic acid; L-165041, [4-[3-(4-Acetyl-3-hydroxy-2-propylphenoxy)propoxy]phenoxy]acetic acid; GW0742, 2-[4-[[2-[3-fluoro-4-(trifluoromethyl)phenyl]-4-methyl-1,3-thiazol-5-yl]methylsulfanyl]-2-methylphenoxy]acetic acid; MHY908, 2-[4-(5-chloro-1,3-benzothiazol-2-yl)phenoxy]-2-methylpropanoic acid; NSAIDs, non-steroidal anti-inflammatory drugs; 1-DNJ, 1-deoxynojirimycin; GST, Gagam-Sipjeondaebo-Tang; PGC-1α, PPAR-gamma co-activator-1 alpha; MPTP, 1-methyl-4-phenyl-1,2,3,6-tetrahydropyridine; 6-OHDA, 6-hydroxyDA; LPS, lipopolysaccharide; MPTPp, probenecid model of Parkinson’s disease; MPP+, 1-methyl-4-phenylpyridinium ion; SH-SY5Y, Human neuroblastoma cells; SN-PC, substantia nigra pars compacta; TH, tyrosine hydroxylase; SN, substantia nigra; GFAP, glial fibrillary acidic protein; DA, dopamine; NF-κB, nuclear factor kappa B; JNK, Jun N-terminal kinase; COX-2, cyclooxygenase-2; iNOS, inducible nitric oxide synthase, NO, nitric oxide; MAPK, mitogen-activated protein kinase; PI3K, phosphoionositide 3-kinase; TNF-α, tumor necrosis factor-α; CNS, central nervous system; Bcl-2, B-cell lymphoma 2; ROS, reactive oxygen species; CAT, catalase; SOD, superoxide dismutase; ER, endoplasmic reticulum; DNA, deoxyribonucleic acid; IL-6, interleukin-6; IL-1β, interleukin-1 beta; PD, Parkinson’s disease; APOD, apolipoprotein D; MITF, microphthalmia-associated transcription factor; TFEB, transcription factor E-box binding.

## Abbreviations

PD, Parkinson’s disease; DA, dopamine; SN-PC, substantia nigra pars compacta; UPS, ubiquitin-proteasome system; PPARs, Peroxisome proliferator-activated receptors; NHR, nuclear hormone receptors; NSAIDs, non-steroidal anti-inflammatory drugs; LBs, lewy bodies; RXR, retinoid X receptors; PPREs, peroxisome proliferator response elements; FA, fatty acids; H_2_O_2_, hydrogen peroxide; N, amino; DNA, deoxyribonucleic acid; C, carboxy; L-165041, [4-[3-(4-Acetyl-3-hydroxy-2-propylphenoxy)propoxy]phenoxy]acetic acid; GW-501516, 2-[2-methyl-4-[[4-methyl-2-[4-(trifluoromethyl)phenyl]-1,3-thiazol-5-yl]methylsulfonyl]phenoxy]acetic acid; TZDs, thiazolidinediones; NF-κB, nuclear factor kappa B; ATF-1, activating transcription factor-1; ATF-4, activating transcription factor-4; STAT, signal transducer and activator of transcription; COX-2, cyclooxygenase-2; NO, nitric oxide; CREB, cyclic AMP-response element binding protein; RELA, REL-associated protein; IκBα, IkappaB alpha; AP-1, activator protein-1; O_2_, oxygen; mtDNA, mitochondrial DNA; Tfam, mitochondrial transcription factor A; PGC-1α, PPAR-gamma co-activator-1 alpha; NT2, neuronal NTERA-2; 2Fe-2S, iron-sulfur; CNS, central nervous system; Bcl-2, B-cell lymphoma 2; Aβ, amyloid-beta; AD, Alzheimer’s disease; HD, Huntington’s disease; ALS, Amyotrophic lateral sclerosis; MS, Multiple sclerosis; LBs, lewy bodies; *UCHL1*, *ubiquitin C-terminal hydrolase L1*; *SNCA*, *α-synuclein*; *LRRK2*, *leucine-rich repeat kinase 2*; *Parkin*, *Parkin RBR E3 ubiquitin-protein ligase*; *PINK1*, *PTEN-induced kinase 1*; *DJ-1*, *protein deglycase*; *GBA*, *glucocerebrosidase*; CH_3_OH, methanol; CO, carbon monoxide; PGP9.5, protein gene product 9.5; gad, gracile axonal dystrophy; PTEN, protein encoded by the *PINK1* gene; GluCer, glucosylceramide; ACC, anterior cingulate cortex; SN, substantia nigra; ALP, autophagy-lysosomal pathway; ER, endoplasmic reticulum; MPTP, 1-methyl-4-phenyl-1,2,3,6-tetrahydropyridine; MPP+, 1-methyl-4-phenylpyridinium ion; Cu, copper; Zn, zinc; Fe, iron; Al, aluminum; Pb, lead; ROS, reactive oxygen species; GSH, glutathione; MDA, malondialdehyde; OGG1, 8-oxoguanine DNA glycosylase; hOGG1, human 8-oxoG DNA glycosylase; hOGG1-2a, hOGG1 type 2a; 8OHG, 8-hydroxyguanosine; LOOH, lipid hydroperoxides; SOD, superoxide dismutase; CAT, catalase; iNOS, inducible nitric oxide synthase; NADPH, nicotinamide adenine dinucleotide phosphate; O_2_^−^, oxygen free radicals; ONOO^−^, peroxy-nitrite radicals; AR, aldose reductase; TH, tyrosine hydroxylase; NO_2_^−^, nitrite; NOx, nitrates; MPO, myeloperoxidase; HOCl, hypochlorous acid; Cl^−^, chloride ions; OH, hydroxyl free radicals; NO_2_^−^, nitrite free radicals; PBMCs, peripheral blood mononuclear cells; PA28, proteasome activator 28; PA700, proteasome activator 700; HSP35, hereditary spastic paraplegia 35; HSC70, heat shock cognate protein 70; LAMP1, lysosomal-associated membrane protein 1; LAMP2A, lysosomal-associated membrane protein 2A; LC3, microtubule-associated protein 1A/1B-light chain 3; KRS, Kufor-Rakeb syndrome; IFNγ, interferon gamma; TNF-α, tumor necrosis factor-α; IL, interleukins; IFN, interferons; NM, neuromelanin; ETC, electron transport chain; TOM20, translocase of the inner mitochondrial membrane 20; MAOB, monoamine oxidase B; GFAP, glial fibrillary acidic protein; 6-OHDA, 6-hydroxyDA; Fis1, mitochondrial fission 1; MFN2, mitochondria fusion 2; NRF1, nuclear respiratory factor 1; NRF2, nuclear respiratory factor 2; LPS, lipopolysaccharide; JNK, Jun N-terminal kinase; PGE2, prostaglandin E2; MAPK, mitogen-activated protein kinase; PI3K, phosphoinositide 3-kinase; MPTPp, probenecid model of Parkinson’s disease; CH_3_CHO, acetaldehyde; NLRP3, nucleotide-binding domain and leucine-rich-repeat-protein 3; GW0742, 2-[4-[[2-[3-fluoro-4-(trifluoromethyl)phenyl]-4-methyl-1,3-thiazol-5-yl]methylsulfanyl]-2-methylphenoxy]acetic acid; MHY908, 2-[4-(5-chloro-1,3-benzothiazol-2-yl)phenoxy]-2-methylpropanoic acid; 1-DNJ, 1-deoxynojirimycin; IL-6, interleukin-6; GST, Gagam-Sipjeondaebo-Tang; IL-1β, interleukin-1 beta; APOD, apolipoprotein D; MITF, microphthalmia-associated transcription factor; TFEB, transcription factor E-box binding; PARIS, parkin-interacting substrate; ZFP, Zn-finger protein; L, ligand; SH-SY5Y, Human neuroblastoma cells; ATP, Adenosine triphosphate; AMPK, AMP-activated protein kinase; Ca^2+^, calcium; ADO, adenosine; HRT, hormone replacement therapy.
